# Diagnosis and Management of Neonatal Hypoglycemia: A Comprehensive Review of Guidelines

**DOI:** 10.3390/children10071220

**Published:** 2023-07-14

**Authors:** Sonia Giouleka, Maria Gkiouleka, Ioannis Tsakiridis, Anastasia Daniilidou, Apostolos Mamopoulos, Apostolos Athanasiadis, Themistoklis Dagklis

**Affiliations:** 1 Third Department of Obstetrics and Gynecology, School of Medicine, Faculty of Health Sciences, Aristotle University of Thessaloniki, Konstantinoupoleos 49, 54642 Thessaloniki, Greece; soniagiouleka@outlook.com (S.G.); daniilidouanastasia@gmail.com (A.D.); amamop@auth.gr (A.M.); apostolos3435@gmail.com (A.A.); tdagklis@gmail.com (T.D.); 2 University College London Hospital, University College London Medical School, 250 Euston Road, London NW1 2PG, UK; mariagkiouleka@outlook.com

**Keywords:** neonatal hypoglycemia, blood glucose levels, plasma glucose, glucose, dextrose, diagnosis, definition, operational threshold, risk factors, clinical signs, screening, management, guidelines, comparison

## Abstract

Hypoglycemia represents one of the most frequent metabolic disturbances of the neonate, associated with increased morbidity and mortality, especially if left untreated or diagnosed after the establishment of brain damage. The aim of this study was to review and compare the recommendations from the most recently published influential guidelines on the diagnosis, screening, prevention and management of this common neonatal complication. Therefore, a descriptive review of the guidelines from the American Academy of Pediatrics (AAP), the British Association of Perinatal Medicine (BAPM), the European Foundation for the Care of the Newborn Infants (EFCNI), the Queensland Clinical Guidelines-Australia (AUS), the Canadian Pediatric Society (CPS) and the Pediatric Endocrine Society (PES) on neonatal hypoglycemia was carried out. There is a consensus among the reviewed guidelines on the risk factors, the clinical signs and symptoms of NH, and the main preventive strategies. Additionally, the importance of early recognition of at-risk infants, timely identification of NH and prompt initiation of treatment in optimizing the outcomes of hypoglycemic neonates are universally highlighted. All medical societies, except PES, recommend screening for NH in asymptomatic high-risk and symptomatic newborn infants, but they do not provide consistent screening approaches. Moreover, the reviewed guidelines point out that the diagnosis of NH should be confirmed by laboratory methods of BGL measurement, although treatment should not be delayed until the results become available. The definition of NH lacks uniformity and it is generally agreed that a single BG value cannot accurately define this clinical entity. Therefore, all medical societies support the use of operational thresholds for the management of NH, although discrepancies exist regarding the recommended cut-off values, the optimal treatment and surveillance strategies of both symptomatic and asymptomatic hypoglycemic neonates as well as the treatment targets. Over the past several decades, ΝH has remained an issue of keen debate as it is a preventable cause of brain injury and neurodevelopmental impairment; however, there is no clear definition or consistent treatment policies. Thus, the establishment of specific diagnostic criteria and uniform protocols for the management of this common biochemical disorder is of paramount importance as it will hopefully allow for the early identification of infants at risk, the establishment of efficient preventive measures, the optimal treatment in the first hours of a neonate’s life and, subsequently, the improvement of neonatal outcomes.

## 1. Introduction

Neonatal hypoglycemia (NH) is the most common neonatal metabolic disturbance [1] and constitutes a leading cause of term admission to neonatal units worldwide [2]. Its incidence is estimated to be 5–15% in otherwise healthy neonates [3,4]. The definition of clinically significant hypoglycemia remains one of the most controversial issues in contemporary neonatology, as blood glucose (BG) concentration is not routinely measured in healthy asymptomatic infants who may experience transient hypoglycemia as part of their normal adaptation to extrauterine life [5]. Thus, the normal range of blood glucose levels (BGL) in the first 48 h of life is yet to be determined [1].

Delayed diagnosis, as well as the suboptimal management of NH, is associated with adverse short- and long-term sequelae in the offspring; acute brain injury, visual-motor impairment, executive dysfunction and neurodevelopmental impairment have been reported [6,7,8]. It is worth noting that despite the fact that several studies and clinical trials have attempted to identify the BGL considered to be safe and to provide a valid estimate of the effect of neonatal hypoglycemia on neurodevelopment [9], evidence from the current literature does not support a specific concentration of BG that can potentially result in acute or chronic irreversible neurologic damage and neither the duration nor the severity of NH can accurately predict permanent neurological damage [6].

Although occasions where NH is severe enough to cause long-term neurodevelopmental harm with subsequent significant costs for the family, the patients and the health systems are rare [10], clinicians should implement practices to prevent harm stemming from failure to recognize or treat NH whilst eliminating unnecessary interventions and admissions to neonatal units and, therefore, avoiding the pointless separation between the mother and the neonate. To date, there is insufficient and inconclusive evidence regarding the definition and treatment protocols of NH, leading to significant discrepancies in the existing guidelines. Thus, the development of international evidence-based algorithms for the early identification, the effective prevention and the successful management of clinically significant low BGL seems to be of insurmountable importance and will hopefully drive favorable neonatal outcomes.

The aim of this descriptive review was to synthesize and compare recommendations from influential guidelines on the diagnosis and management of neonatal hypoglycemia.

## 2. Evidence Acquisition

The most recently published guidelines by influential medical societies on NH were retrieved and a descriptive review was conducted. In particular, six guidelines were identified from: the American Academy of Pediatrics (AAP 2011) [11], the British Association of Perinatal Medicine (BAPM 2017) [12], the European Foundation for the Care of the Newborn Infants (EFCNI 2018) [13], the Queensland Clinical Guidelines-Australia (AUS 2019) [14], the Canadian Pediatric Society (CPS 2020) [15] and the Pediatric Endocrine Society (PES 2015) [16].

An overview of recommendations is presented in Table 1 (risk factors and clinical signs of NH) and Table 2 (screening, diagnosis and management of NH), respectively. Of note, five of the reviewed guidelines focus mostly on the transitional NH in the immediate postnatal period; however, the recommendations made by PES mainly address the subject of persistent NH, including the diagnosis and management of disorders causing recurrent or prolonged hypoglycemia that persists or occurs beyond the first 72 h of life.

## 3. Definition of Neonatal Hypoglycemia

Many healthy infants experience transient hypoglycemia as part of their normal adaptation to extrauterine life, resulting from the discontinuation of nutrients due to the separation from the placental circulation [5]. This leads to a transient reduction in BGL beginning at 1 to 2 h after birth, known as “physiologic” hypoglycemia (as low as 30 mg/dL (1.6 mmol/L) according to the AAP and BAPM or 20–25 mg/dL (1.1–1.4 mmol/L) according to EFCNI and AUS). The lowest point is usually reached in the first 2 to 4 h of life; at 4 to 6 h, the BGL usually stabilize at 2.5–4.4 mmol/L (45–79 mg/dL) [17]. Glucose is the major oxidative fuel of the brain; however, this transient, asymptomatic form of hypoglycemia can be relatively easily compensated through the production of alternative sources of energy, such as ketone bodies released from fat. After the first 2 postnatal hours, the glucose concentration begins to rise, mainly due to endogenous production (glycogenolysis and gluconeogenesis) rather than feeding. This is the result of a mild and transient form of hyperinsulinism where the mean threshold of BGL for the suppression of insulin secretion is lower in newborn babies (55–65 mg/dL (3.0–3.6 mmol/L)) than in older infants and children (80–85 mg/dL (4.4–4.7 mmol/L)) [18]. The mechanism responsible for the glucose-stimulated insulin secretion matures with age, resulting in an increase in the mean threshold of BGL, which, by 72 h of age, is similar to those in older infants and children [18].

It is common for healthy, breast-fed newborns to present low BGL (<36 mg/dL (2 mmol/L)) during the first 24 h of life [19] without abnormal clinical signs or symptoms. A randomized controlled trial, called “The Sugar Babies Study”, which enrolled 514 infants of 35–42 gestational weeks, younger than 48 h old, identified to be at risk for NH, found that 51% of babies became hypoglycemic (BGL < 47 mg/dL (2.6 mmol/L)) and 19% had severe hypoglycemia (BGL < 36 mg/dL (2.0 mmol/L)). The majority of the hypoglycemic ones, i.e., 79%, showed no clinical signs [3]. Given these facts, defining a clinical diagnosis of NH is crucial to provide guidance for when and whether therapy should be initiated.

If any infant shows clinical manifestations compatible with significantly low BGL, such as apnea, jitteriness and seizures, the plasma glucose (PG) or BG concentration should be measured immediately. The AAP and PES support measuring PG levels to define hypoglycemia, while the BAPM, EFCNI, AUS and CPS recommend whole BGL measurement. PG values tend to be higher compared to the whole blood glucose levels by approximately 10–18% (AAP), 10–15% (BAPM, EFCNI), 15% (PES), 10% (CPS), because the concentration of water in the plasma is higher than in the whole blood [18].

However, the definition of NH lacks uniformity among the reviewed guidelines. First, although all societies divide newborns into two groups depending on their postnatal age, to make a distinction between transient and persistent NH, the AUS, BAPM and PES use a cutoff of 48 h, while CPS and EFCNI draw the line at 72 h of age. The PES guideline are based not only on the neonate’s age but also on the presence or absence of a known or suspected hypoglycemic congenital disorder, as they mostly address the matter of evaluation and management of persistent NH. Furthermore, the CPS recommends a different cutoff of glucose levels in transient (within the first 72 h of life) than in persistent NH (beyond the first 72 h of life), as the former is defined by BGL lower than 2.6 mmol/L (47 mg/dL) (also endorsed by AUS and AAP), while the latter by BGL lower than 3.3 mmol/L (59 mg/dL). The definition of persistent NH given by the EFCNI is consistent, i.e., NH lasting beyond 72 h of postnatal life. In contrast, the BAPM guidelines define transient NH (during the first 48 h of life) by BGL between 1.0 and 1.9 mmol/L (18–34 mg/dL) documented on one or two occasions, whereas persistent NH (beyond the first 48 h of life) is defined by BGL lower than 2.0 mmol/L (36 mg/dL) on more than two occasions. The AUS and PES also propose the cut-off point of 48 h to distinguish transitional from persistent hypoglycemia and the AUS describes recurrent NH as BGL below 2.6 mmol/L (47 mg/dL) on more than three occasions in a row.

The definition of severe NH is also controversial. More specifically, the BAPM mentions that NH should be characterized as severe when BGL are <1.0 mmol/L (18 mg/dL), while the AUS suggests a definition of BGL < 1.5 mmol/L (27 mg/dL), BGL not recordable or symptomatic hypoglycemia.

This distinction has implications on management as transient NH in the absence of associated clinical manifestations does not require further investigation [20], while severe and persistent NH should prompt urgent medical attention and additional investigations because it may be the first sign of a severe metabolic disorder, like hyperinsulinemic hypoglycemia or hypopituitarism [21].

On the other hand, the term “clinical hypoglycemia” is used by the PES and AUS guidelines to describe the concentration of PG that is low enough to cause brain injury [22].

## 4. Screening for Neonatal Hypoglycemia

There is no consensus regarding the exact timing when screening should be performed (AAP). Data regarding both the optimal timing and time intervals for screening blood glucose are limited and it remains controversial whether it is necessary to screen the at-risk newborns who do not present any signs or symptoms of NH during the time that BGL reach their normal lowest point (approximately within 1–2 h after delivery) [23]. Furthermore, the evidence supporting routine screening for NH of asymptomatic infants who have no risk factors for hypoglycemia, after a non-complicated pregnancy and delivery, is insufficient.

Five of the reviewed guidelines (AAP, BAPM, EFCNI, AUS, CPS) provide guidance for the screening of NH. They all agree that screening for NH should be performed only for infants with suspected or well-established risk factors for developing hypoglycemia; any infant with abnormal feeding behavior, absence of feeding cues or any other clinical manifestations should be promptly screened for NH at any time; in fact, screening is recommended within minutes, not hours, of the appearance of symptoms and with a duration and frequency of BGL testing that depend on individualized risk factors.

With regard to the initial screening, BAPM, EFCNI and AUS support that the optimal time for screening of asymptomatic, at-risk neonates is just before the second feed (practically no longer than 2–4 h after delivery) provided that the newborn is offered feeding within the first hour after birth. On the contrary, according to AAP and CPS, the recommended time for screening high-risk infants is 30 min after the first feed (practically up to 2 h of age) followed by intervention with feeding or IV glucose depending on the glucose values. The AAP and CPS agree with the BAPM and AUS on the timing of the initial feed, which should be offered to the neonate within the first hour after delivery. Regarding the subsequent BGL measurements, after the initial screening of asymptomatic at-risk infants, all five medical societies agree that measurements should be performed prior to feedings. Breast milk or formula feedings should be offered to newborns every 2–3 h or more frequently.

Furthermore, the AUS and BAPM guidelines suggest a second BGL screening before the third feed and no later than six hours (AUS) or eight hours (BAPM) of age. However, the subsequent steps differ. More specifically, according to the AUS, if BGL is within the normal range (≥2.6 mmol/L, >47 mg/dL), screening should continue to be performed before every second feed (every three to six hours depending on feeding frequency) for 24 h. On the contrary, if the second BGL measurement is above 2.0 mmol/L, the BAPM proposes no further glucose measurements, unless signs or symptoms indicative of hypoglycemia are present, and only recommends observation for 24 h, providing continuous support of breastfeeding. According to the CPS, testing should also be performed one or two times during the second day of life, to ensure that the BGL remain above 2.6 mmol/L (47 mg/dL), whereas the AAP suggests repeated testing prior to feedings after the first 24 h of age only if PG values remain lower than 45 mg/dL (2.5 mmol/L).

Additionally, the AAP and the CPS agree upon continuing measurements through multiple feed–fast cycles depending on the risk factors of each newborn. On the one hand, small-for-gestational-age (SGA) and late-preterm neonates should be screened for at least the first 24 h before each feeding (every 2–3 h); in addition, if the BGL remain above 2.6 mmol/L (47 mg/dL), screening should be discontinued [24]. On the other hand, large-for-gestational-age neonates and those of diabetic mothers should be screened only for the first 12 h after birth, with the same cut-off glucose value used for discontinuing measurements. This difference in the duration of BGL screening is based on studies showing that IDM and LGA infants are more likely to become hypoglycemic by 12 h after the birth, in contrast to preterm and SGA infants, who usually develop asymptomatic NH within 24 h [24,25,26].

## 5. Diagnosis of Neonatal Hypoglycemia

Diagnosing NH using a single glucose value is neither feasible nor simple [19]. Thus, monitoring, managing and preventing NH remain highly pressing issues [27]. According to the AUS, CPS and AAP, the generally adopted PG concentration cut-off for otherwise healthy infants is 47 mg/dL (2.6 mmol/L). More specifically, the CPS guideline refers to the existence of four approaches to the diagnosis of NH based on the following aspects: 1. the neonate’s clinical condition; 2. epidemiological data from studies on exclusively breastfed, appropriate-for-gestational-age (AGA), term infants and their measured BGL [4,21,28]; 3. the presence or absence of normal physiological responses to NH; and 4. the presence or absence of brain injury and long-term sequelae.

However, as stated by AAP, there is no robust scientific justification for the generally adopted cut-off of blood glucose for NH in all infants (47 mg/dL, 2.6 mmol/L) [23,28] and the normal range of blood glucose concentration in neonates depends on various factors, such as their birthweight, gestational age, clinical manifestations, energy sources and metabolic demands. The reasons that make it difficult to form and adopt a substantial, evidence-based definition for NH and an accurate value for BG that requires intervention in all neonates are the frequent co-existence of other severe medical conditions and the lack of evidence on the levels of BG and the duration of NH that can cause brain injury and long-term neurological sequelae, alone or in concert with comorbidities [4,22].

This is why the approach of the “operational threshold” has been introduced by a panel of experts that convened in 2000 [4] and has been endorsed by all six medical societies to guide interventions intended to restore BGL. An operational threshold constitutes the concentration of BGL (either plasma or whole blood) that should raise awareness of physicians to consider intervention based on evidence available in the current literature, distinguishing between the BG value that requires action and the target BGL that interventions aim for [4]. This “operational threshold” approach has been widely adopted for all neonates at risk of impaired metabolic adaptation and adverse outcome, but the threshold values for whole BG or PG for diagnosis of NH and consequent intervention remain a matter of keen debate.

Thus, according to BAPM, the most important threshold concentrations at which clinicians should consider intervention include: 1. a BG value < 1.0 mmol/L (<18 mg/dL) at any time, 2. a single value < 2.5 mmol/L (45 mg/dL) in a neonate with abnormal clinical signs, and 3. a value < 2.0 mmol/L (36 mg/dL) that remains that low in a subsequent measurement, in case of a newborn with one risk factor for impaired metabolic adaptation but not presenting any abnormal clinical signs and/or symptoms. These thresholds are higher when it comes to symptomatic newborn infants with recurrent or persistent hyperinsulinemic hypoglycemia (HH). In such cases, therapeutic levels of 3.0 mmol/L (54 mg/dL) or more are suggested [12]. According to AUS, any neonate with BGL < 1.5 mmol/L or unrecordable measurement, as well as any symptomatic neonate, requires urgent management and further investigation, while the value used as an operational threshold is BGL below 2.6 mmol/L (47 mg/dL) in all at risk neonates. The PES recommends PG levels to be kept >2.8 mmol/L (50 mg/dL) during the first 48 h of postnatal life and >3.3 mmol/L (60 mg/dL) after 48 h for high-risk neonates without a suspected congenital hypoglycemic disorder. The same operational threshold for blood glucose but in a different time window (after 72 h of life) is recommended by the CPS guidelines, while for the first 72 h postpartum, the CPS suggests the threshold glucose value of 2.0 mmol/L, for which further management is required. The PES recommend that the operational threshold for neonates with a suspected congenital or confirmed hypoglycemic disorder is higher, as in such cases the PG must be maintained >70 mg/dL (3.9 mmol/L), in contrast with 3.0 mmol/L suggested by the BAPM and 3.3 mmol/L by the AUS. Moreover, PES defines the considered-to-be-normal PG values for neonates as 55–65 mg/dL in the first 48 h of age and 70–100 mg/dL for older ones. The AAP recommends operational thresholds for PG concentration in high-risk newborns that differ depending on the hours of age: 25–40 mg/dL (1.4–2.2 mmol/L), 35–45 mg/dL (1.9–2.5 mmol/L) and 45 mg/dL (2.5 mmol/L), from birth to 4 h of life, from 4–24 h of life and after 24 h of life, respectively. The AAP also recommends intervention for all neonates with clinical signs and a PG concentration less than 40 mg/dL. Finally, the EFCNI, adopts the operational threshold approach on guiding interventions and clinical decisions based on glucose values approved by professionals in all maternity and neonatal units; however, they underline the profound controversy among recommendations of different organizations, due to the lack of evidence-based data on cerebral damage provoked by NH [29]. Thus, the EFCNI does not specifically define NH, only stating that BGL as low as 1.0 mmol/L (18 mg/dL) are associated with acute neurological impairment [9,23].

## 6. Diagnostic Methods of Neonatal Hypoglycemia

The accurate measurement of BGL is crucial for the diagnosis and treatment of NH. Therefore, the optimal methods of BGL assessment are discussed in all guidelines reviewed. Blood glucose levels are usually measured using chemical strips or bedside handheld glucose meters (non-enzymatic methods) and most of the time they are not validated using laboratory diagnostic tests [15,30].

However, the accuracy of bedside reagent test-strip glucose analyzers is limited, especially in the low range of BG concentrations. This low range is defined as 10–15 mg/dL (0.6–0.8 mmol/L) by the PES, and as 0–36 mg/dL (0–2.0 mmol/L) by the BAPM and EFCNI, whereas no specific values are provided by the other societies. It is also crucial to keep in mind that the neonatal packed cell volume (PCV) could be a cause of inaccuracy in hand-held glucometers due to the fact that they do not auto-correct for this variable. Samples with high PCV can generate falsely low glucose values and vice versa [12]. Moreover, even though only few devices that measure true whole BG values by rupturing red blood cells are available, most handheld test-strip glucometers report results that demonstrate a reasonable correlation with PG concentrations and that are considered to be “PG equivalents”. Whole BG and PG levels may vary up to 10 to 20 mg/dL, but the gap becomes wider at low glucose concentrations.

These are the reasons why these point of care methods are not reliable enough to be used as the sole method for NH screening [30,31], as highlighted by all six guidelines. More specifically, the AAP, PES, CPS and AUS guidelines state that the initial screening could be performed using “rapid” bedside tests (including handheld reflectance colorimeter and electrode methods validated for neonatal samples), to prevent any delay for the rapid diagnosis and initiation of treatment, provided that the clinician is aware of their limited accuracy. Capillary samples obtained from a warmed heel can be used for screening, as agreed by all these guidelines.

However, due to the limitations of these handheld glucometer devices, before establishing a diagnosis of NH, glucose concentration (plasma or whole blood) must be confirmed using laboratory enzymatic methods (glucose oxidase, hexokinase and dehydrogenase methods). According to AAP, although not rapidly available, laboratory testing is the most accurate method for BGL measuring. The AUS specifies that, if the initial screening of BGL is <2.6 mmol/L (47 mg/dL) in neonates with clinical manifestations compatible with hypoglycemia or with risk factors for NH, glucose values should be validated using point-of-care diagnostic tests (such as enzymatic handheld glucometers with glucose oxidase or glucose dehydrogenase methodology, if available), blood gas analyzers or laboratory enzymatic methods (in fluoride oxalate tube, if feasible to be performed immediately). The same diagnostic methods are recommended by the AUS, in case of initial BGL < 2.0 mmol/L (36 mg/dL), in all newborn infants. As delineated by the AUS, AAP and CPS guidelines, treatment should not be delayed while waiting for the results to be confirmed using a laboratory test, especially for severe, persistent or recurrent NH [4]. Additionally, the CPS guideline mentions another diagnostic method for NH, called CGMs (continuous glucose monitors), which, however, have numerous limitations that question their accuracy; the development of other more promising and more accurate point-of-care devices for bedside glucose measurement may improve the screening methods for NH. On the contrary, the BAPM and EFCNI state that blood gas analyzers are quick, widely available and accurate for measuring BG values. Furthermore, they calculate glucose result as “PG equivalent” concentration, which in most cases is similar to the result obtained from a laboratory enzymatic diagnostic method. Thus, blood gas biosensors are considered to be the gold standard in the screening of NH, as they support real-time clinical decision making and they could be set up to provide a ‘glucose only’ reading on a tiny neonatal blood sample [32]. If handheld glucometers are used (necessarily compliant with the specific ISO15197:2013 standard), it is highly important for clinicians to remember their limitations in accuracy at low BGL and to confirm their results with more accurate techniques to ensure that hypoglycemic infants are assigned to the optimal care pathway. As stated by the BAPM, a laboratory confirmation may not be practical, not only because of the delay in obtaining results but also due to inconsistency of the results, caused by variability in the inhibition of glycolysis in fluoride oxalate tubes. Lastly, a new technology—currently under development—based on transdermal, minimally invasive, constant and accurate blood sugar measurements provided by biosensors is discussed in the BAPM guidelines as a very promising useful tool for future research [33].

## 7. Prevention

There is general agreement on the basic principles of NH prevention among the BAPM, EFCNI, AUS and CPS guidelines. These include the following: 1. the antenatal or immediate postnatal identification of all at-risk infants; 2. the avoidance of cold stress and hypothermia—ideally by providing skin to skin contact with the mother; 3. the early and timely energy provision and feeding support; 4. the regular BGL monitoring at predetermined times with accurate devices that provide results with no delay; 5. the constant observation of both the feeding behavior and the overall clinical condition of the neonate; and 6. a thorough discussion with the parents regarding the neonate’s feeding and well-being. The BAPM, EFCNI and AUS guidelines describe these principals in detail. On the other hand, the AAP does not mention any measures for the prevention of NH, the CPS focuses on the neonate’s feeding standards to prevent NH, and the PES only refers to disorders with persistent NH, such as hyperinsulinism, in which the main goal of prevention is trying to avoid recurrent episodes of hypoglycemia that may increase the risk of subsequent, possibly unrecognized hypoglycemic episodes.

Clinicians should keep in mind that early recognition is vital to avoid serious health disorders and improve outcomes. First, the risk factors for NH must be identified at birth to provide meticulous care and extra support to the newborns. More specifically, the AUS highlights that preterm infants of ≤35 gestational weeks should be admitted to neonatal units and receive special care by managing other possible co-existing clinical conditions, ensuring thermal care and providing early and frequent feeds, assisted with gavage if needed or indicated for neonates not nippling well (AAP, AUS, BAPM).

Additionally, a thorough and regular assessment of the neonate’s clinical condition when awake is important. The general appearance, muscle tone, body measurements, body malformations or deformations (indicative of a syndrome potentially responsible for NH), skin color, body temperature (normal range within 36.5–37.5 °C measured via the axilla), level of consciousness, response to external stimulations, respiratory and heart rate and all feeding cues should be evaluated [10]. Abnormal feeding behaviors that should raise awareness and call for action include not waking for meals, not latching at the breast, not sucking effectively and appearing unsettled. The BAPM and AUS guidelines point out that when signs or symptoms suggestive of NH make their appearance, BGL should be immediately measured and a pediatrician or a neonatal nurse practitioner should be called for assistance and further guidance.

Moreover, the BAPM, EFCNI and AUS thoroughly describe all the steps that should be followed to prevent hypothermia of the at-risk neonate, including the use of a hat, the avoidance of cold draughts, the warmth of the ambient temperature and the immediate skin-to-skin contact with the mother, while the CPS suggests that the first bath should be delayed for at-risk infants as it has been found to decrease the incidence of NH [34].

The crucial role of the parents in the monitoring and management of infants at risk for impaired metabolic adaptation is highlighted by three of the reviewed guidelines (BAPM, CPS and EFCNI). They point out that parents should participate actively in the care pathway of at-risk neonates, being aware not only of the reasons behind their newborns’ requirement of extra care and why they undergo regular blood testing for measuring BGL, but also of all the signs and symptoms that could indicate hypoglycemia. Thus, parents can learn about the importance of early energy provision and help physicians with BG monitoring. If risk factors for NH are known before delivery, health care providers should communicate with the parents to inform them antenatally. The BAPM suggests that this information should be given to parents in both verbal and written form, while the EFCNI suggests giving this information only verbally.

The BAPM, EFCNI, AUS and CPS note that breast milk is the optimal source of energy for all neonates during their postpartum metabolic adaptation. The early initiation of feeds plays a significant role in preventing NH and it should be ensured that the neonate is offered the breast within the first 60 min (BAPM) or 30–60 min (AUS) of life [10,35]. Efficient support should be provided to all mothers to make them feel capable of initiating and establishing effective breastfeeding and to enable them to recognize both early feeding cues and signs of effective attachment. Feeding effectiveness should be assessed at each feed and the breastfeeding should be offered at least 8–10 times in 24 h, according to feeding cues. As stated by the BAPM, there should not exist a gap of more than three hours between the meals until BGL exceeds 2 mmol/L (36 mg/dL) on two or more consecutive measurements [12]. The main goal is to cover the neonate’s energy demands as much as possible using breast milk or expressed colostrum/breast milk.

In formula-fed infants, the timing of initial feed and time intervals between feedings are practically the same. The AUS guideline supports that complementary feeds are not required in the first 24 h of life, unless one BGL measurement is <2 mmol/L (36 mg/dL) or two or more BGL values are <2.6 mmol/L (47 mg/dL), whereas it mentions that if formula feeding is chosen, meals should be up to 60–75 mL/kg/day for at-risk newborns. In cases where complementary feeds are required, a minimum of 7.5 mL/kg/feed should be provided [10]. Τhe CPS guidelines differ in that they suggest supplementing feeds with breast milk or a breast milk substitute; the total volume of both oral and IV intake should not exceed 100 mL/kg/day so as to avoid fluid overload and serum electrolytes disorders. This medical society also highlights the importance of continuing to feed high-risk infants regularly, while continuing to measure BGL prior to meals, as well as the use of a pump to achieve slow feeding (breast milk or formula) rather than bolus feeding.

## 8. Management of Asymptomatic Neonatal Hypoglycemia

The goals of managing NH are as follows: first, to identify at risk newborns and newborns with serious underlying hypoglycemic disorders [36]; second, to correct BGL; and third, to avoid unnecessary treatment of normal transitional NH, which will likely resolve without intervention [37]. It is crucial to keep in mind that the treatment of hypoglycemia is a stepwise process depending on the presence or absence of symptoms and signs and on the infant’s response at each step. All of the reviewed guidelines highlight the importance of recognizing and treating asymptomatic NH early and agree on the main principles of management, which are as follows: 1. the antenatal or immediate postpartum identification of risk factors, 2. the provision of thermal care, 3. the early energy provision and feeding support, 4. the regular monitoring of BGL and infusion of IV dextrose when necessary, and 5. to try not to interrupt the mother–infant relationship and breastfeeding when possible.

For asymptomatic newborns at risk, the AAP suggests a treatment plan that is divided into two time periods, up to 4 h of age and between 4 and 24 h of age. An initial feed should be offered to all neonates within the first hour of age and an initial screen of BGL should be performed 30 min after the first feed. If the PG is <25 mg/dL (1.3 mmol/L), another feeding-checking PG in a one hour-cycle is recommended, and if PG remains <25 mg/dL, IV glucose administration is indicated (glucose dose 200 mg/kg, 2 mL/kg dextrose 10% D/W). If the PG is between 25 and 40 mg/dL (1.3–2.2 mmol/L), another attempt to feed may be made before progressing with glucose administration [38]. For newborns aged 4 to 24 h, feeding every 2–3 h (after the initial feed) and PG measurements prior to each feed are recommended. If PG is <35 mg/dL (1.9 mmol/L) in one sample, it is suggested to refeed and recheck PG concentration within 1 h. If PG remains <3 5 mg/dL, intravenous glucose should be administered (same dose as before). However, if PG is between 35 and 45 mg/dL (1.9–2.5 mmol/L), active support of feeding should continue before the initiation of treatment with IV dextrose solution.

According to the BAPM and AUS guidelines, at-risk neonates should be placed in two care pathways based on their first pre-feed BGL. For the BAPM, the first cut-off point is BGL between 1.0 and 1.9 mmol/L (18–34 mg/dL) in infants with no abnormal clinical signs, while the second cut-off point is either BGL < 1.0 mmol/L (18 mg/dL) in neonates without clinical manifestations or higher BGL but with neonates showing symptoms consistent with NH. For the AUS, the cut-off points are as follows: 1. BGL between 1.5 and 2.5 mmol/L (27–45 mg/dL) in asymptomatic neonates; and 2. BGL below 1.5 mmol/L (27 mg/dL) or unrecordable values or symptomatic neonates within the first 48 h of life.

The BAPM suggests that when BGL are between 1.0 and 1.9 mmol/L (18–34 mg/dL) and no clinical manifestations are present, the administration of 40% oral dextrose gel (dose of 200 mg/kg) should be considered as part of the feeding plan, alongside breastfeeding or formula feeding, if the mother chooses so. The AUS recommendations for at-risk asymptomatic infants with BGL 1.5–2.5 mmol/L (27–45 mg/dL) and the CPS recommendations for at-risk infants with BGL < 2.6 mmmol/L (47 mg/dL) agree with those of the BAPM, as a dose of 40% dextrose gel is suggested to be given buccally (dose of 0.5 mL/kg equivalent to 200 mg/kg) in conjunction with oral feedings. The EFCNI also aligns with the aforementioned guidelines on this matter, as it is generally stated that oral dextrose gel may be considered as an adjunct to a feeding plan in high-risk newborns. This oral 40% dextrose gel of 0.5 mL/kg provides a dose of 200 mg/kg glucose, which is equivalent to the intravenous bolus glucose dose of 2 mL/kg of the 10% DW solution. Its administration is indicated only in late preterm and term infants (CPS) or neonates > 35 weeks of gestational age (BAPM, AUS) during the first 48 h after delivery, with a maximum of six doses during this period of time (AUS, BAPM). The “Sugar Babies” study, which is described in the CPS and BAPM guidelines, assessed the effectiveness of dextrose oral gel treatment over feeding alone in hypoglycemic neonates and showed that therapy with dextrose gel leads to significant lower treatment failure rates compared to placebo. The buccal gel has also been found to reduce the number of NICU admissions due to NH, alongside the need for supplementation with formula at 2 weeks of age [39]. In fact, if glucose gel administration is followed by immediate breastfeeding, the quality of subsequent breast feeds is improved [40]. However, although it decreases the need for IV glucose administration, it cannot achieve the complete avoidance of IV therapy [39].

Furthermore, according to the BAPM, BG should be measured again prior to the third feed and no longer than 8 h of age, and if BGL fail to rise above 2 mmol/L (36 mg/dL), another circle of oral dextrose gel and feeding should be repeated. A re-check of BGL is also recommended by the AUS (30 min after the first dose of oral dextrose gel) and a subsequent dose of dextrose gel is considered safe to be administered if the BGL remain between 2.0 and 2.5 mmol/L (36–45 mg/dL). Similarly, according to CPS, BGL should be re-measured 30 min post-feed and if they remain between 1.9 and 2.6 mmol/L (34–47 mg/dL), another loop of oral dextrose gel 40% (same dosage) followed by enteral supplementation (breastfeeding or formula feeding) and a glucose measurement again 30 min after feeding is recommended. On the contrary, if BGL are <1.9 mmol/L (34 mg/dL) (CPS), 1.0 mmol/L (18 mg/dL) (BAPM) or 1.5 mmol/L (AUS), the initiation of IV glucose infusion at hourly requirements (10% DW) is strongly advised without repeating the loop of oral dextrose gel–breastfeeding/formula feeding/EBM.

In addition, if more than two measurements between 1.0 and 1.9 mmol/L have been documented or if two consecutive doses of glucose gel 40% have been given, the neonatal team should be informed to investigate possible causes of NH and to exclude other disorders that mimic hypoglycemia, like sepsis. Admission to the Neonatal Intensive Care Unit (NICU) is required (BAPM, AUS) in such cases. An increase in the feeding frequency and the insertion of a nasogastric tube should also be considered and the IV glucose administration (10% DW) at this point is suggested, too. It is important to remember that buccal dextrose gel can be used as first-line treatment for hypoglycemia, allowing the infant–mother relationship not to be interrupted, avoiding NICU hospitalization and improving the chances of effective breastfeeding after discharge [39].

Additionally, as stated by the BAPM, if BGL are >2.0 mmol/L, breastfeeding or formula feeding and/or EBM should continue to be offered, glucose should be measured again prior to the next feed, and if BGL remain >2.0 mmol/L (after two consecutive pre-feed BG measurements) and no clinical manifestations are present, it is advised that BG measurements are discontinued. According to the AUS, the conditions under which cessation of BGL monitoring is indicated are as follows: (a) BGL ≥ 2.6 mmol/L or ≥3.3 mmol/L for 24 h, within or beyond the first 48 h of life, respectively, (b) neonate feeding effectively, (c) asymptomatic neonate for whom IV glucose had not been required. For neonates who were treated with IV dextrose but are now feeding well and have not received IV glucose during the past 12 h, monitoring should be ceased when BGL exceed 3 mmol/L for two successive measurements. CPS suggest ceasing pre-feed glucose monitoring when two consecutive BG samples are above 2.6 mmol/L and the neonate fully tolerates enteral feeds.

## 9. Management of Symptomatic Neonatal Hypoglycemia

The appearance of hypoglycemic clinical signs and symptoms constitutes a red flag for the urgent initiation of therapy because severe, prolonged, symptomatic hypoglycemia may result in neuronal injury [38,41]. First, a laboratory confirmation of the low BGL must always be performed before starting IV treatment, according to the AAP, BAPM and AUS, because it is essential for both the identification and the optimal management of hypoglycemia. However, therapy should not be delayed while waiting for laboratory results. Blood samples during the hypoglycemic period should be collected to perform further diagnostic evaluation [42].

The recommendations of AAP in symptomatic infants with BGL < 40 mg/dL (2.2 mmol/L) involve immediate IV glucose treatment either as an IV bolus glucose dose of 200 mg/kg (2 mL/kg 10% DW) or as an IV glucose infusion of 80–100 mL/kg 10% DW per day to maintain PG concentrations between 40 and 50 mg/dL (2.2–2.7 mmol/L). The CPS guideline agrees with this approach of immediately treating symptomatic infants or infants who cannot be orally fed, with an IV infusion of 10% DW or a bolus IV glucose administration (dose of 2 mL/kg over 15 min) when BGL are lower than 1.8 mmol/L. The administration of a bolus dose at the start of a glucose infusion therapy is believed to stabilize BGL more rapidly. The PES instructions also align with this treatment for any episode of severe symptomatic hypoglycemia with IV dextrose infusion at an initial dose of 200 mg/kg, followed by infusion of 10% DW at a maintenance rate. A response to the intravenous administration of glucose is expected in the next 30 min and a confirmation should be performed in a timely manner [43].

The recommendations of EFCNI and BAPM on symptomatic hypoglycemia or newborns presenting with very low glucose levels (<1.0 mmol/L, 18 mg/dL) are consistent, as they suggest that in such cases infants should be treated with IV glucose as an initial bolus of 2.5 mL/kg 10%DW (instead of 2 mL/kg 10%DW) as soon as possible, followed by a glucose infusion administration of 60 mL/kg 10% DW per day (instead of 80–100 mL/kg/day). The recommended of the AUS for initial IV bolus glucose dose for symptomatic newborns or BGL below 1.5 mmol/L (27 mg/dL) is 1–2 mL/kg 10% DW, followed by the re-measurement of BGL in the next 30 min and repeated by another bolus glucose dose of 1 mL/kg IV while monitoring for rebound hypoglycemia. The IV glucose infusion rate should commence at 60 mL/kg/day 10% DW. The AUS also gives instructions for treating newborns with BGL between 1.5 and 2.5 mmol/L who are not feeding well (symptomatic newborns). In such cases, one dose of 40% oral dextrose gel should be given, a neonatal nurse practitioner or a pediatrician should be informed, a lactation consultant should be notified and BGL should be re-measured within 30 min. If the BGL are between 2.0 and 2.6 mmol/L, a second dose of 40% oral dextrose gel can be administered and breastfeeding or formula feeding and/or EBM should be continued. If the BGL are <2 mmol/L, the neonate must be admitted to the NICU in order to initiate IV treatment.

There is a consensus among the reviewed guidelines that for the management of symptomatic NH, an intravenous access should be obtained (peripheral or central). The AUS points out that in case the required IV glucose infusion concentration is more than 12%, an umbilical venous catheter or central line should be inserted; however, the CPS question previous data that dictated the need for a central vein for glucose solutions with concentration ≥ 15% and supports the integrity of peripheral veins with dextrose concentrations up to 20% based on a randomized controlled trial of 121 hypoglycemic newborns, which showed that 20% and 15% glucose solutions can be infused equally safely into peripheral veins in neonates [44]. Nevertheless, in case an IV access is not easy or possible to be established immediately, two alternatives are proposed as urgent interventions: 40% dextrose gel 200 mg/kg equivalent to 0.5 mL/kg- administered orally via buccal massage (BAPM), or intramuscular injection of glucagon 200 microgram/kg (BAPM, AUS, CPS). It is important, however, to keep in mind that if the BGL are <1.0 mmol/L, the buccal dextrose gel should only be used as an interim measure while trying to establish an IV line [45].

The continuation of treatment is based on the regular assessment of the neonatal clinical condition and its BGL monitoring. The PES, AAP and EFCNI guidelines do not discuss in detail the next steps of the neonate’s ongoing management, whereas the BAPM, AUS and CPS recommendations agree that if the first intervention is followed by failure to raise BGL, a stepwise increase in glucose supply may be necessary. The AUS recommends that the glucose rate should be daily increased by 20 mL/kg, without exceeding the total daily fluid intake of 100 mL/kg on the first day of life, to prevent fluid overload. The concentration of the IV dextrose solution could also be increased from 10% DW to 12% or higher, keeping in mind the necessity to always measure BGL after any changes to glucose concentration. The same applies to the increase in the glucose delivery rate proposed by BAPM (mentioned as a rise of 2 mg/kg/min) either by increasing the volume or the concentration of IV dextrose solution. At this point, these medical societies agree that if the glucose infusion rate (GIR) is higher than 8 mg/kg/min in the first 24 h after delivery (or, according to BAPM, if BGL is <2.0 mmol/L on more than two measurements during the first 48 h of life), a clinical suspicion of hyperinsulinism should be raised and treatment with glucagon should be commenced. BGL should be measured again in the next 30 min.

According to the BAPM, if the BGL remain <1.0 mmol/L or there are abnormal clinical signs, another cycle of treatment should be repeated with IV bolus 10% DW (2.5 mL/kg), followed by an increase in the glucose infusion delivery rate and re-measurement of BGL 30 min afterwards. If the BGL are between 1.0 and 2.5 mmol/L with no abnormal clinical manifestations, the GIR is suggested to increase by 2 mg/kg/min without another IV bolus dextrose administration, and feedings should continue unless there are contraindications. If the BGL are >2.5 mmol/L, a slow and gradual weaning of IV infusion should start and the enteral feeds should also continue. It is necessary to continue BGL monitoring until the infant is on full enteral feeds and the BGL are >2.5 mmol/L (or 3.0 mmol/L in cases of hyperinsulinism) for several fast–feed cycles during the first 24 h of life.

## 10. Alternative Treatments

The use of alternative medications for the management of NH in cases where BGL do not become normal after the administration of IV glucose or 40% buccal dextrose gel is addressed by the CPS, PES, BAPM and AUS guidelines. The decision for a long-term therapy for hypoglycemic disorders (either persistent or recurrent) should be made in consultation with an experienced neonatologist, a pediatric endocrinologist or a pediatric metabolic specialist in cases where either glucose infusion rate is very high (>10 mg/kg/min according to CPS or >8 mg/kg/min according to the AUS) or glucose infusions fail to maintain the BGL at acceptable levels (more than two blood sugar measurements of 1.0–1.9 mmol/L during the first 48 h postnatally according to the BAPM; greater than 2.6 mmol/L up to 48 h of age or 3.3 mmol/L after the first 48 h, according to the AUS). Blood samples for further investigations (such as serum cortisol and insulin) should be collected immediately while the newborn remains hypoglycemic before administering any medications because recurrent or persistent NH may be the first sign of an underlying disorder associated with the metabolism of glucose, such as hyperinsulinism, disorders leading to cortisol and growth hormone deficiency and inborn errors of metabolism [42,46]. Regarding these alternatives to glucose administration, the AUS and CPS suggest the utilization of glucagon, hydrocortisone, diazoxide and octreotide, while the AUS also proposes hydrochlorothiazide and the BAPM only mentions glucagon as an alternative when an IV line is difficult to be accessed. On the other hand, PES discourages non-specific treatment with glucocorticoids for NH and recommends the use of glucagon, surgical intervention and nutritional therapies.

Glucagon stimulates gluconeogenesis and glycogenolysis and it can result in raising BGL in term and preterm hypoglycemic infants (AUS, PES, CPS). The CPS guideline states that glucagon may be given via IV bolus or infusion, whereas the AUS, BAPM and PES point out that an intramuscular or subcutaneous injection could be considered—apart from IV administration—if it is not possible or easy to establish an IV access [47]. The IV infusion of glucagon is preferred by the AUS because it prevents an exaggerated stimulation of the pancreas due to a high glucose infusion rate and it does not interfere with the effective establishment of breastfeeding. Additionally, the AUS does not align with the PES regarding the onset of action and duration of glucagon, as the former supports that the BGL rise within one hour upon administration and last, approximately, up to two hours [47], while the latter indicates that the BGL increase within 10–15 min and remain at these levels for at least 1 h. Hypoglycemia non-responsive to glucagon may be provoked by glycogen storage disease [48].

Moreover, hydrocortisone is proposed as an alternative treatment for NH by the AUS and CPS because its mechanism of action includes the stimulation of gluconeogenesis and the reduction in glucose utilization in peripheral tissues. It is remarkable that hydrocortisone has a slower response than glucagon [49]. Hydrocortisone may be preferred when hyponatremia is suspected, the infant is hypotensive, evidence indicative of hypoadrenalism is present or the response to previously administered glucagon is insufficient.

Diazoxide is a potassium channel activator used in cases of persistent NH as long-term management. Its mechanism of action is the inhibition of pancreatic insulin release and can be used in conjunction with hydrochlorothiazide in order to achieve weaning from glucose infusion. Hydrochlorothiazide (proposed as an alternative treatment by the AUS) is a diuretic, which has a mechanism of action similar to the one of diazoxide.

Octreotide is a pharmacological analog to natural somatostatin, usually recommended for known or suspected cases of hyperinsulinemic hypoglycemia, and not indicated for the newborn period.

When medical therapy fails to maintain the BGL in a safe range, surgical intervention is proposed by the PES for neonates with hyperinsulinemic hypoglycemia. The importance of nutritional therapy is emphasized by the PES, especially for disorders of glycogen metabolism or hereditary fructose intolerance.

Although it is not a pharmacological intervention, the AUS describes the increase in fluid volume as an effective alternative measure to manage severe, persistent or recurrent NH. Increasing the volume of IV glucose prior to increasing the concentration of glucose to 12% will result in an immediate change in glucose delivery rate whilst a solution of increased glucose concentration is prepared. In particular, a rise of 20 mL/kg/day in the total volume fluids (which does not exceed the maximum daily fluid intake) leads to an approximate 33% increase in BGL. The maximum tolerated total fluid intake is 100 mL/kg/day for most babies of less than 24 h of age, without being at risk of fluid overload. Serum electrolytes should be monitored within regular intervals in order to avoid hyponatremia and over-hydration.

## 11. Target Glucose Concentration and Discharge Plan

The reviewed guidelines, based on the physiology of normal neonatal glucose homeostasis, the normal age-related increase in glucose concentrations over the first few days of life, and the various pathophysiological conditions that may result in clinical hypoglycemia recommend steps of treatment in order to initiate therapy in a timely manner and to avoid the complications of NH. This treatment is a long process that depends on BG or PG measurements, the presence or absence of symptoms and/or signs and the infant’s clinical response, too. Glucose target values vary among these guidelines, alongside with the discharge criteria of at-risk neonates.

The AAP recommends that the target PG concentration should be >45 mg/dL (2.5 mmol/L) pre-prandially and that neonates should be capable of maintaining normal PG values throughout at least three feed–fast periods of time. The BAPM suggests that the therapeutic goal should be a BGL value > 2.0 mmol/L (36 mg/dL). The AUS states that the BGL target for neonates younger than 48 h of age is >2.6 mmol/L (47 mg/dL) for three feed–fast cycles, while for those older than 48 h with a known hypoglycemic disorder, the target is >4.0 mmol/L (72 mg/dL) for three feed–fast cycles. The CPS supports that the BGL target for newborns younger than 72 h should be >2.6 mmol/L (47 mg/dL) and for newborns older than 72 h > 3.3 mmol/L (60 mg/dL). Finally, the PES states that neonates with a suspected hypoglycemic congenital disorder, as well as older infants and children, should have BGL > 70 mg/dL (3.9 mmol/L) to achieve the therapeutic goal. For high-risk neonates without a congenital hypoglycemic disorder, the target value of PG is >50 mg/dL (2.8 mmol/L) or >60 mg/dL (3.3 mmol/L) for those up to 48 h of age and for those older than 48 h, respectively. The therapeutic target for glucose levels is not discussed by the EFCNI.

With regard to the discharge plan, the BAPM and EFCNI agree that newborns should not be discharged until at least two consecutive pre-prandial glucose measurements are within the normal range and neonates have been feeding effectively over several fast–feed cycles. BAPM clarifies that pre-feed BG measurements should be >2.0 mmol/L for neonates with initial BGL measurements between 1.0 and 1.9 mmol/L and no clinical signs, and >2.5 mmol/L (or 3.0 mmol/L) for neonates with initial BGL below 1.0 mmol/L with/without clinical signs in order to cease monitoring. The AAP states that neonates should maintain normal PG concentrations for at least three feed–fast periods before discharge. The AUS aligns with the recommendations of PES on the management and follow- up of neonates (older than 48 h of age) with a known or suspected cause of persistent or prolonged hypoglycemic disorder or with clinically significant NH (requiring a GIR > 6 mg/kg/min or medication such as diazoxide or hydrochlorothiazide), proposing a safety test of six hours of fasting with regular BG measurements in the interval. This fasting test should be performed after consultation with a pediatric endocrinologist or metabolic specialist and should take place before discharge from nursery to ensure that high-risk neonates are capable of remaining normoglycemic if a feeding is missed, as well as to identify infants who need further investigation and additional management for a persistent hypoglycemic disorder.

## 12. Conclusions

To summarize, there is an overall agreement among the reviewed guidelines regarding the risk factors associated with NH, the wide variety of non-specific clinical manifestations and the main principles of NH prevention. All medical societies underline that the timely identification of hypoglycemic neonates and immediate initiation of treatment are crucial in preventing permanent brain injury. In addition, the AAP, BAPM, EFCNI, AUS and CPS recommend screening for NH using BG measurement for all symptomatic neonates as well as for all asymptomatic high-risk ones. The diagnosis of NH should be confirmed via laboratory tests; however, a single BG value cannot accurately define NH. Thus, all guidelines endorse the “operational threshold approach” for the management of subsequent interventions.

On the other hand, there is inconsistency concerning the screening algorithms, the definition of NH, the threshold values of glucose for the diagnosis of NH and the treatment protocols of asymptomatic hypoglycemic newborns. Minor discrepancies were also identified regarding the initial intravenous bolus dose of glucose, the following rate of continuous infusion and the alternative therapies of symptomatic neonates as well as the treatment targets. It should be noted that one of the major limitations of this descriptive review, which may partially explain the inconsistency identified across the different medical organizations, is that NH represents a complex condition which may occur due to a variety of causes.

The controversy of the guidelines regarding the management of NH and the lack of universal applicability due to inconsistent definitions and the paucity of a substantial body of evidence is clearly outlined. However, NH remains one of the most common and severe metabolic disturbances in perinatal medicine, with destructive consequences when left untreated. This descriptive review attempts to distill the burgeoning literature and place emphasis on the importance of adopting and implementing consistent international protocols for the definition, diagnosis, operational thresholds, prevention and treatment of NH, with the goal of assisting healthcare providers in best managing hypoglycemic neonates and subsequently minimize the rates of associated neonatal morbidity and mortality. New evidence is constantly being published and the understanding of NH is evolving; further large-scale randomized studies are required to validate and modify the diagnostic and therapeutic approaches suggested by the guidelines.

## Figures and Tables

**Table 1:** Risk factors and clinical signs of neonatal hypoglycemia.

	AAP	BAPM	EFCNI	AUS	CPS	PES
Country	United States	United Kingdom	Europe	Australia	Canada	International
Issued	March 2011	April 2017	November 2018	September 2019	December 2020	August 2015
Title	Clinical Report—Postnatal Glucose Homeostasis in Late-Preterm and Term Infants	Identification and Management of Neonatal Hypoglycemia in the Full Term Infant	Hypoglycemia in at-risk term infants	Hypoglycemia–newborn	The screening and management of newborns at risk for low blood glucose	Recommendations from the Pediatric Endocrine Society for Evaluation and Management of Persistent Hypoglycemia in Neonates, Infants and Children
Pages	7	35	8	38	17	8
References	31	81	24	75	75	39
Risk factors	SGA, LGA, maternal diabetes, late prematurity	FGR, maternal diabetes, maternal beta-blockers in 3rd trim +/− at delivery), moderate to severe perinatal hypoxia-ischemia, suspected/confirmed early onset sepsis, pituitary/adrenal insufficiency, inborn errors of metabolism, hyperinsulinism, family history of 1st degree relative with a heritable hypoglycemic disorder.	FGR, maternal diabetes, asphyxia, maternal beta-blockers, sepsis, hemolytic disease, specific inborn errors of metabolism, congenital disorders that prevent infants from mounting an adequate counter-regulatory metabolic and endocrine response.	FGR, LGA, macrosomia, maternal medication, maternal diabetes, hyperinsulinemia, family history of genetic form of hypoglycemia or congenital hyper-insulinemic or endocrine disorder, sibling or parent with MCADD, PE/eclampsia or GH or other placental insufficiency, intrapartum IV glucose > 20 g/h, neonate’s T < 36.5 °C, perinatal asphyxia, PTL or postmature, neonate with seizures, delayed/inadequate feeding, IV therapy–abrupt cessation or rapid weaning of glucose, meconium aspiration, polycythemia, hypothyroidism, severe hepatic dysfunction, erythroblastosis, inborn errors of metabolism.	SGA, FGR, LGA, maternal diabetes, prematurity maternal labetalol use, late exposure to antenatal steroids, perinatal asphyxia, metabolic conditions, syndromes associated with hypoglycemia.	FGR, LGA, SGA, perinatal stress/asphyxia/ischemia, PE/eclampsia or GH, meconium aspiration, erythroblastosis, polycythemia, hypothermia PTL or postmature delivery, maternal diabetes, family history of a genetic form of hypoglycemia, congenital syndromes, abnormal physical features (midline facial malformation, microphallus).
Clinical signs of NH	Jitteriness, cyanosis, seizures, apneic episodes, tachypnea, weak or high-pitched cry, floppiness or lethargy, poor feeding, eye-rolling. Coma and seizures if prolonged and repetitive NH.	Perinatal acidosis, T < 36.5 °C, early onset sepsis, cyanosis, apnea, altered level of consciousness, seizures, hypotonia, lethargy, high-pitched cry, abnormal feeding especially after a period of feeding well, jitteriness.	Abnormal feeding	Apnea, bradycardia, cyanosis, tachypnea, hypothermia, jitteriness, persistent tremor, irregular breathing, sweating, irritability, pallor, poor feeding, hypotonia, abnormal cry, seizures, changes in level of consciousness–stupor, coma, lethargy, apathy.	Jitteriness or tremors, cyanosis, convulsions, intermittent apneic spells or tachypnea, weak or high-pitched crying, limpness or lethargy, abnormal feeding, eye-rolling, sweating, sudden pallor, hypothermia, cardiac arrest and failure.	Palpitations, tremor, anxiety, sweating, hunger, paresthesia, confusion, coma and seizures.

LGA: large-for-gestational-age; SGA: small-for-gestational-age; FGR: fetal growth restriction; MCADD: medium chain acyl-CoA dehydrogenase deficiency; PE: preeclampsia; GH: gestational hypertension; T: temperature; PTL: preterm labor; IV: intravenous; NH: neonatal hypoglycemia.

**Table 2:** Summary of recommendations on screening, diagnosis and management of NH.

	AAP	BAPM	EFCNI	AUS	CPS	PES
Country	United States	United Kingdom	Europe	Australia	Canada	International
Issued	March 2011	April 2017	November 2018	September 2019	December 2020	August 2015
Title	Clinical Report—Postnatal Glucose Homeostasis in Late-Preterm and Term Infants	Identification and Management of Neonatal Hypoglycemia in the Full Term Infant	Hypoglycemia in at risk term infants	Hypoglycemia–newborn	The screening and management of newborns at risk for low blood glucose	Recommendations from the Pediatric Endocrine Society for Evaluation and Management of Persistent Hypoglycemia in Neonates, Infants and Children
Pages	7	35	8	38	17	8
References	31	81	24	75	75	39
Screening for NH	Recommended in term infants with clinical signs or at risk. PG (within minutes, not hours) if clinical signs of low BGL. Frequency and duration individualized. At-risk infants should be fed by 1 h of age and screened 30 min later. After 24 h, repeat before feedings if PG remains <45 mg/dL.	Recommended if abnormal clinical signs, reluctant/non-effective feeding after a period of effective feeding, infants not effectively fed. Optimal time to measure BGL: prior to second feed (<4 h of delivery). If no feeding cues within 4 h, measure BG.	Recommended before the 2nd feed and no later than 4 h after birth in asymptomatic infants, or at any time if abnormal clinical signs.	Screening times: 1st BGL before 2nd feed and <3 h of age. 2nd BGL screen before 3rd feed and <6 h of age. If normal (≥2.6 mmol/L), screen before every 2nd feed–every 3–6 h pre-feed for 24 h.	Recommended for asymptomatic, at-risk infants at 2 h of age and 30 min post feed. When 2 consecutive samples are >2.6 mmol/L, continue monitoring pre-feed or every 3–6 h. Symptomatic and unwell infants require immediate glucose testing. Screen once or twice on day 2 when more than one PG < 2.6 mmol/L in the first 24 h.	Not discussed
Diagnosis-Operational Thresholds	PG concentration defining NH for all infants < 47 mg/dL. Operational thresholds: 25–40 mg/dL (1.4–2.2 mmol/L) in first 4 h, 35–45 mg/dL (1.9–2.5 mmol/L) from 4–24 h and 45 mg/dL (2.5 mmol/L) after 24 h of life.	BGL < 1.0 mmol/L at any time (severe hypoglycemia). A single BGL < 2.5 mmol/L if abnormal clinical signs. BGL < 2.0 mmol/L and remaining < 2.0 mmol/L at next measurement in at-risk baby, without clinical signs. Persistent hypoglycemia: ≥3 measurements < 2.0 mmol/L in the first 48 h. Consider hyperinsulinism if BGL remain low (<2.0 mmol/L on ≥3 occasions in first 48 h), or if glucose dose > 8 mg/kg/min required. BGL threshold 3.0 mmol/L if suspected hyperinsulinism < 48 h after birth.	BGL < 1.0 mmol/L (18 mg/dL) associated with acute neurological dysfunction present the greatest risk of cerebral injury.	NH definition: symptomatic baby and/or BGL < 2.6 mmol/L. Severe hypoglycemia: BGL < 1.5 mmol/L. Prolonged hypoglycemia: >48 h. Recurrent hypoglycemia: ≥3 sequential episodes of BGL < 2.6 mmol/L.	Transitional hypoglycemia < 72 h post-birth: BGL < 2.6 mmol/L. Persistent hypoglycemia: BGL < 3.3 mmol/L > 72 h post-birth. Threshold BGL that requires action: 2.0 mmol/L.	Normal PG > 48 h: 70–100 mg/dL (3.9–5.5 mmol/L). Normal PG < 48: >55–65 mg/dL (3.0–3.6 mmol/L). In suspected congenital hypoglycemia disorder and older infants and children with a confirmed hypoglycemia disorder, treatment target recommended: PG > 70 mg/dL (3.9 mmol/L). For high-risk neonates without a suspected congenital hypoglycemia disorder, treatment target suggested: PG > 50 mg/dL (>2.8 mmol/L) < 48 h and >60 mg/dL (>3.3 mmol/L) > 48 h.
Diagnostic methods	Laboratory enzymatic methods (Glucose oxidase, hexokinase or Dehydrogenase). Consider bedside reagent test-strip glucose analyzers (handheld reflectance colorimeter and electrode methods) if test performed carefully and clinician aware of their limited accuracy.	Ward-based blood gas biosensor (reference standard for measuring BG). Hand-held glucometers (prone to limited accuracy particularly in the range 0–2.0 mmol/L, -only use if ISO15197:2013 standard).	Ward-based blood gas biosensor (reference standard for measuring BG). Hand-held glucometers conforming to the ISO 15197:2013 standard (inaccurate particularly in the range 0–2.0 mmol/L).	Point of care glucometer with enzymatic methods (glucose oxidase or dehydrogenase). Otherwise, a calibrated non-enzymatic glucometer with electrochemical sensor validated for neonatal samples (may be unreliable at lower BGLs). If screening BGL < 2.6 mmol/L or borderline in a neonate at risk or with clinical signs of hypo-glycemia: Validate by diagnostic test using point of care analyzer, blood gas analyzer or laboratory specimen in fluoride oxalate tube.	While acute management can be initiated based on point-of-care samples to prevent delay, a diagnosis of persistent hypoglycemia should be confirmed by laboratory assays. Continuous glucose monitors (CGMs) of questionable accuracy.	Clinical laboratory method. Point-of-care meters: convenient screening method but with limited accuracy. Before establishing a diagnosis of NH, essential to confirm low PG by a clinical laboratory method.
Prevention	Not discussed	Keep neonate dried and warm with hat and blanket after birth. Skin-to-skin contact with the mother. Encourage early breast feeding within the 1st hour after birth. Not without meal for >3 h. Regular neonatal assessment when awake (color, tone, RR, HR, T, level of consciousness and signs of hypoglycemia).	Thermal care with skin-to-skin contact. Support breast feeding and discuss feeding cues. Early energy provision. Monitoring of BGL starting within the first hours of life.	Assess for RF. Keep baby warm and dried. Maintain T 36.5–37.5 °C. Early to skin contact. Initiate feeds within 30–60 min of birth. Feed at least three times hourly or more frequently. Formula feed if maternal choice or with consent if breast milk not available (at risk baby: 60–75 mL/kg/day as tolerated). If baby < 35 w admit to NICU. If clinical condition allows, early—frequent feeds.	Increase breastfeeding frequency. Supplement feeds with breast milk or breast milk substitute. Slow feeding with breast milk or formula using a pump rather than bolus feeding. Increase carbohydrate intake. Delay the first bath.	For disorders such as hyperinsulinism, aim to prevent recurrent hypoglycemia that increases the risk of subsequent, possibly unrecognized, hypoglycemic episodes.
Management of asymptomatic NH	Asymptomatic at-risk infants should be fed by 1 h of age and screened 30 min later. If BGL < 25 mg/dL (<4 h of age) or <35 mg/dL (4–24 h of age), refeed and recheck BG 1 h later. Subsequent BGL < 25 mg/dL, or <35 mg/dL, respectively, after attempts to refeed, necessitate IV glucose treatment.	Neonates with pre-feed BG 1.0–1.9 mmol/L and no abnormal clinical signs or neonates with subsequent BGL < 2.0 mmol/L, should be treated with 40% buccal dextrose gel 200 mg/kg. Support breast feeding. If BGL ≥ 2.0 mmol/L, breastfeed and/or offer expressed breast milk. For formula fed infants give 10–15 mL/kg in 3 hourly feed volumes. If BGL < 2 mmol/L before the 3rd feed repeat one loop of 40% buccal dextrose gel 200 mg/kg.	Consider oral dextrose gel as an adjunct to a feeding plan in newborn infants at risk of hypoglycemia.	If BGL is 1.5–2.5 mmol/L and baby is ≥35 w, well and feeding, give oral glucose gel 40% and ensure that the baby has an effective feed (feed at least 3 hourly). If BGL 2–2.6 mmol/L, administer a 2nd dose of oral glucose gel 40%. If BGL is <1.5 mmol/L admit the baby to neonatal unit and start IV glucose 10%.	Give 40% dextrose gel 0.5 mL/kg or feed 5 mL/kg and breastfeed. Check glucose 30 min post-feed. To augment caloric intake and before starting IV dextrose, provide enteral supplementation for asymptomatic infants (BGL: 1.9–2.6 mmol/L).	Not discussed
Management of symptomatic NH	Prompt intervention required. Obtain plasma sample for a laboratory glucose determination just before giving an IV “minibolus” of glucose (200 mg of glucose/kg, 2 mL/kg dextrose 10% in water IV) and/or starting a continuous infusion of glucose (D_10_W at 80–100 mL/kg/day).	If BGL < 1.0 mmol/L and/or clinical signs of NH, obtain IV access, give IV 10% glucose 2.5 mL/kg, start IV infusion of 10% glucose at 60 mL/kg/d. Do not stop breast feeding unless baby too sick to feed or contraindication to enteral feeding. In formula-fed infants, continue feeds if no contraindication. Recheck BGL after 30 min.	If clinical signs or very low BGL, IV dextrose (IV bolus of 2.5 mL/kg 10% glucose) as soon as possible, followed by constant rate glucose infusion.	Not discussed	Administer IV dextrose if not responded to enteral supplementation. If neurological signs, treat immediately with an IV infusion of glucose. Response to IV glucose rechecked after 30 min. If failure to respond, stepwise increase in glucose supply, with a review of levels 30 min after each increment.	IV dextrose infusion. Initial dose: 200 mg/kg, followed by infusion of 10% dextrose at a maintenance rate for age.
Alternative treatments	Not discussed	If unable to obtain immediate IV access, give 40% dextrose gel 200 mg/kg massaged into the buccal mucosa while IV access is obtained or IM glucagon (200 mg/kg).	Not discussed	If BGL not normal after buccal glucose gel 40% or IV glucose, consider: Glucagon (in hyperinsulinemic conditions refractory to IV glucose infusion), hydrocortisone, diazoxide, hydrochlorothiazide, octreotide.	When infusions fail to maintain BG at appropriate levels or an especially high rate (>10 mg/kg/min) of infusion is required, consider further investigation/specialist referral, and/or pharmacological intervention (Glucagon, hydrocortisone, diazoxide, octreotide)	Medications for hyperinsulinism, and cortisol or growth hormone deficiency. Consider surgery for hyperinsulinemic children unable to maintain safe BGL through medical therapy. Nutritional therapy for disorders of glycogen metabolism or hereditary fructose intolerance. Some milder disorders may be treated by avoidance of prolonged fasting.
Target Glucose concentration- Discharge plan	Target PG ≥ 45 mg/dL prior to routine feeds. Ensure maintenance of normal PG concentrations on a routine diet for a reasonably extended period (through at least three feed–fast periods) before discharge.	BGL ≥ 2.0 mmol/L. Recommended operational threshold: 3.0 mmol/L in neonates with suspected hyperinsulinism < 48 h after birth. Ensure maintenance of BGL > 2.0 mmol/L on ≥2 consecutive occasions as well as effective feeding before discharging babies at risk.	Not discussed	Discharge if baby < 48 h of age and pre-prandial BGL > 2.6 mmol/L for three feed–fast cycles or if known hypoglycemic condition and baby ≥ 48 h of age and pre-prandial BGL is >4 mmol/L for three feed–fast cycles. A 6 h fast test performed (if indicated) and baby able to maintain BGL.	Target BGL > 2.6 mmol/L for babies younger than 72 h of age and BGL > 3.3 mmol/L for older ones.	Target PG > 70 mg/dL (3.9 mmol/L) for neonates with a suspected congenital hypoglycemic disorder and older infants and children with a hypoglycemic disorder. Target PG > 50 mg/dL (2.8 mmol/L) for high-risk neonates without suspected congenital hypoglycemic disorder aged <48 h and PG > 60 mg/dL (3.3 mmol/L) for those aged >48 h.

NH: neonatal hypoglycemia; PG: plasma glucose; BGL: blood glucose levels; RR: respiratory rate; HR: heart rate; RF: risk factors; T: temperature; NICU: neonatal intensive care unit; IV: intravenous; IM: intramuscular; BG: blood glucose.

## Data Availability

Data are publicly available.
